# The Attitude of Egyptian Patients with Autoimmune and Rheumatic Diseases towards Telemedicine

**DOI:** 10.3390/medicina59091595

**Published:** 2023-09-04

**Authors:** Samar Tharwat, Doaa Gamal

**Affiliations:** 1 Rheumatology & Immunology Unit, Department of Internal Medicine, Faculty of Medicine, Mansoura University, Mansoura 35511, Egypt; 2 Department of Internal Medicine, Faculty of Medicine, Horus University, New Damietta 34517, Egypt; 3 Mansoura Manchester Programme for Medical Education, Faculty of Medicine, Mansoura University, Mansoura 35511, Egypt; www.doaagamal70@gmail.com

**Keywords:** telemedicine, COVID-19, rheumatic diseases, autoimmune diseases

## Abstract

*Background and Objectives*: The use of telemedicine (TM) has recently undergone rapid growth and proliferation. Professional stakeholders anticipate that TM will aid in the efficient allocation of limited resources in rheumatology care. The aim of the study was to evaluate the acceptance and willingness of Egyptian patients with autoimmune and rheumatic diseases (ARDs) to incorporate TM into rheumatological care and to assess their requirements and concerns regarding TM. *Materials and Methods*: A cross-sectional questionnaire-based study was conducted among Egyptian patients with ARDs. The questionnaire covered sociodemographic characteristics, clinical and therapeutic data, attitudes, barriers, and motivators towards TM. *Results*: The study included 189 patients with ARDs, with a mean age of 37 years (SD  =  11.71), and 88.4% were females. Participants were divided into two groups based on their acceptance of TM: the non-acceptant group (133, 70.4%) and the acceptant group (56, 29.6%). There was a significant difference in educational level (*p* = 0.001), chronic kidney and heart disease (*p* = 0.008 and 0.014, respectively) and hydroxychloroquine administration (*p* = 0.037) between the two groups. During the coronavirus disease 2019 (COVID-19) pandemic, 96 (50.8%) of participants used virtual rheumatology consultations, mainly using WhatsApp (64.6%). Approximately 87% would require assistance in operating TM technology. The preference for direct conversation with the rheumatologist and the need for physical examination were the main barriers to teleconsultation. *Conclusions*: TM is opposed by the vast majority of Egyptian patients with ARDs. They are concerned since it does not include a physical examination and prevents them from undergoing additional procedures such as ultrasound and blood testing. The majority of Egyptian patients with ARDs need help using TM technology, which is the most significant barrier to the spread of TM.

## 1. Introduction

The World Health Organization (WHO) declared the coronavirus disease 2019 (COVID-19) outbreak a pandemic on 11 March 2020 [1]. The pandemic has created global upheaval in many sectors of life; it has also overwhelmed health systems [2]. Because of lockdowns and stay-at-home strategies, significant changes have occurred in the utilization of healthcare services [3]. Furthermore, the medical system became overburdened, lowering the standard of service for all individuals seeking medical care, including those with autoimmune and rheumatic diseases (ARDs) [4]. The COVID-19 pandemic has also had a detrimental influence on the healthcare provided to Egyptian patients with ARDs, who have suffered drug shortages as well as hospital admission difficulties [5].

Telemedicine (TM) is defined as the delivery of healthcare services, where distance is a critical factor, by all healthcare professionals using information technologies for the exchange of valid information for the diagnosis, treatment, and prevention of disease and injuries; research and evaluation; and continuing education of healthcare providers, all in the interests of advancing the health of individuals and communities [6]. The COVID-19 pandemic has drastically altered outpatient healthcare delivery. The pandemic disrupted unnecessary in-person outpatient appointments, resulting in a substantial increase in remote diagnostic and treatment services (e.g., TM) for patients with chronic disorders, including those with ARDs [7]. So, the COVID-19 pandemic accelerated and broadened the use of TM in rheumatology care [8]. The applicability of TM for providing care while promoting social distancing to slow disease transmission has resulted in substantial change among stakeholders [9]. The application of TM technology will become increasingly important in the delivery of future medical treatment, patient consultations, and a wide variety of additional expanded uses for healthcare in rural and remote areas [10].

During the pandemic, TM established itself as a viable and more popular technique of providing medical consultation [11] with a positive acceptability of TM as a method of providing care for patients with connective tissue diseases [12,13]. In an online survey conducted in January 2021, a total of 596 individuals diagnosed with ARDs participated. The findings of the study indicate that a significant proportion of respondents expressed that TM is comparably beneficial to in-person consultations, particularly in relation to specific indications [14]. Nevertheless, a study conducted on patients with hidradenitis suppurativa suggests that this approach may not yield desirable outcomes for those who require traditional consultations, as this is the preferred mode of consultation for this specific patient population [15].

Professional stakeholders anticipate that TM will aid in the efficient allocation of limited resources in rheumatology care [16]. TM in rheumatology, also known as telerheumatology, was formerly and primarily utilized to extend care provision in rural and remote areas [8]. Telerheumatology is a relatively new study field, with the majority of available evidence published within the last 20 years. However, the number of publications has significantly increased in recent years, particularly during the COVID-19 pandemic [17].

Telerheumatology is expected to take over a significant portion of rheumatology consultations in the near future [18]. It appears to be a promising alternative for facilitating diagnosis and long-term care. However, little is known about the level of its utilization and perception among populations in developing countries like Egypt [19].

The aim of this study was to evaluate the acceptance and willingness of Egyptian patients with ARDs to incorporate TM into rheumatological care and to assess their requirements and concerns regarding it.

## 2. Materials and Methods

### 2.1. Study Design and Settings

This cross-sectional questionnaire-based study was carried out during the period from 20 June to 15 August 2022 at the Rheumatology and Immunology Unit in Mansoura University Hospital. The main population of interest was patients with ARDs. The study included consecutive adult patients with the diagnosis of ARDs who visited the unit (outpatient or inpatient). Patients aged less than 18 years or who had severe neurological or psychiatric disorders were excluded from the start. All participants were provided with detailed information about the study, and informed written consent was obtained from all of them. For those participants who were illiterate, informed consent for their participation in the study was obtained from a parent or other legal guardian.

### 2.2. Ethical Consideration

This study was carried out in accordance with the principles of the Helsinki Declaration [20]. The Institutional Research Board of Mansoura University’s Faculty of Medicine gave its approval to the study protocol (approval registration number: R.22.11.1963).

### 2.3. Sample Size and Sampling Procedure

Patients with ARDs were asked to participate in the study. Convenience sampling was used to collect data. This sample size calculation was conducted based on G*Power, the outcome of interest was acceptance of TM among patients with ARDs and was 78% [12], the effect size was 0.1, the alpha error was 0.05, and the power of the study was 0.9. So, the sample size was found to be 123 subjects.

### 2.4. Survey

The researchers created the questionnaire after conducting a thorough literature review. The questionnaire was written in English and then translated into Arabic. Following editing and review, five rheumatology staff members evaluated the questionnaire design, quality, language, and simplicity of completion as part of a pilot study that validated the questionnaire. The questionnaire comprised multiple-choice questions. It was tested on 20 patients with ARDs in a pilot study; these patients were later omitted from the data analysis.

### 2.5. Survey Administration

The research was based on interviews. Each interview was supposed to take between 10 and 15 min to finish. The interviewer spoke with as many ARD patients as feasible. During their hospital visits, all patients with ARDs who volunteered to participate were interviewed. A single interviewer performed face-to-face structured interviews with each participant. This style of questioning makes it easier to investigate more complex subjects than self-administered modes of questioning because the interviewer can provide more thorough explanations of questions. The confidentiality and anonymity of the participants were ensured by not requesting any personal information.

### 2.6. Variables Recorded

To adapt the questionnaire to the context of our study, we included significant items based on existing literature findings. Among the studied variables are the following: the questions addressed detailed sociodemographic characteristics such as gender, age, marital status, residence, socioeconomic status, level of education, employment status, and smoking habit. Participants were also asked about their ARDs’ clinical and therapeutic data, such as diagnosis, time since diagnosis, self-reported disease severity, and drugs used for treatment.

Furthermore, the questionnaire included questions about the distance between the patient’s home and the nearest rheumatology outpatient clinic (OPC), the frequency of visits to the rheumatology OPC in the previous period, the number of specialists the patient frequently visited, and the exemption from insurance copayment, which could be complete, partial, or none.

COVID-19 infection clinical data were collected and included the following items: (1) ever being infected with COVID-19; (2) appointment with a virtual rheumatologist during the COVID-19 pandemic; and (3) virtual rheumatology appointment platform: phone, WhatsApp, chat, video conference, or email.

Furthermore, the participants were asked if they preferred TM or in-person visits for rheumatological consulting. Several questions were asked regarding what participants appreciated best about having direct contact with their rheumatologist during a traditional OPC consultation. Other information was gathered regarding the issues related to teleconsultations with rheumatologists and the counsel participants preferred to obtain via teleconsultation for their medical conditions.

Finally, the participants were asked if they needed assistance in participating in the teleconsultation, how they found out about it, and whether they wanted the teleconsultation to continue after the COVID-19 pandemic.

### 2.7. Statistical Analysis

The Statistical Package for Social Science (SPSS) program version 22 was used for data analysis. Quantitative data for parametric variables were given as means with standard deviations (SD) and median (min–max) for nonparametric variables, while qualitative data were expressed as percentages and numbers. The Shapiro–Wilk test was employed to determine the normality of the variable distribution. The significance of differences between two groups was evaluated using the independent-sample t test for normally distributed data and the Mann–Whitney test for nonparametric variables. Chi-square or Fisher exact tests were used to compare qualitative variables, as appropriate. A *p* value of less than 0.05 was considered significant.

## 3. Results

The study was carried out on 189 patients with ARDs (response rate, 75.6%); their mean age was 37 years (SD = 11.71). Most of them were females (88.4%). Approximately 70% were married, and about two-thirds (66.7%) were from urban areas. Participants were divided into two groups based on their acceptance of teleconsultation: the non-acceptant group (133, 70.4%) and the acceptant group (56, 29.6%). Gender, age, marital status, socioeconomic status, and smoking habit were not significantly different between the two groups. However, there was a considerable difference in educational level between the two groups (*p* = 0.001), as shown in Table 1.

The clinical and therapeutic data of the study ARD patients are presented in Table 2. Of them, forty-five (23.4%) were diagnosed with rheumatoid arthritis (RA), 39 (20.6%) with systemic lupus erythematosus (SLE), and 30 (15.9%) with osteoarthritis (OA). The median disease duration was 48 months (1–360) (min–max). Approximately half of them (46.6%) reported moderate disease activity. Associated comorbidities included mainly hypertension (31.7%), chronic pulmonary disease (15.9%), and chronic kidney disease (11.1%). Corticosteroids (51.9%) were the most used therapeutic agents, followed by hydroxychloroquine (49.2%) and methotrexate (23.8%). There was a significant difference between the non-acceptant and acceptant groups regarding chronic kidney and heart disease (*p* = 0.008 and 0.014, respectively) and hydroxychloroquine administration (*p* = 0.037).

Table 3 depicts the healthcare services provided to study participants. We noticed that 78.3% of patients lived in another region away from the OPC, 55.6% of patients visited the rheumatologist every <4 months, and 63.5% of patients had no exemption from insurance copayments. Approximately half of the patients had previously been infected with COVID-19. There was no significant difference between the non-acceptant and acceptant groups in terms of distance from OPC, frequency of rheumatologist visits, exemption from insurance copays, or prior COVID-19 infection.

**Figure 1:**
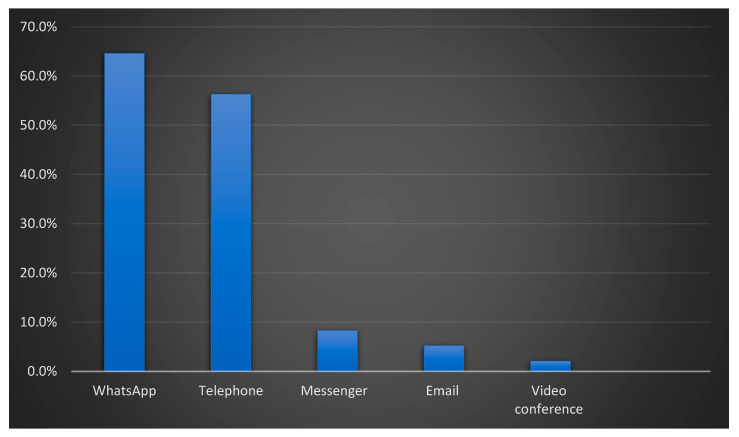
Platform used for virtual rheumatology appointment(s) (n = 96).

During the COVID-19 pandemic, 96 (50.8%) of participants used virtual rheumatology consultations; 64.6% of them utilized WhatsApp, 56.3% used the phone, 8.3% used messenger, and only 2.1% used video conference as virtual appointment platforms as shown in Figure 1.

**Figure 2:**
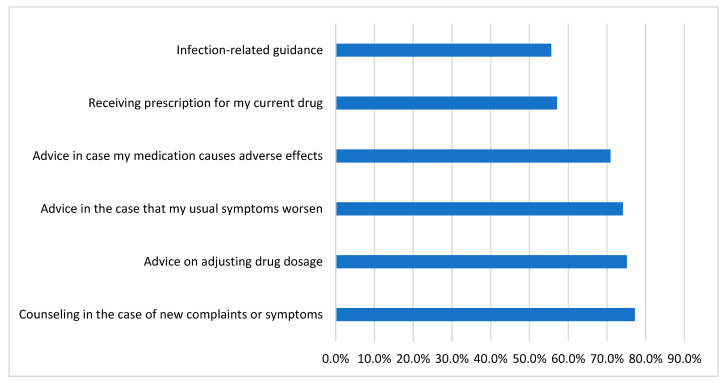
Medical conditions related to rheumatic disease for which participants would like to receive teleconsultation advice (n = 189).

Figure 2 depicts rheumatic-disease-related medical disorders for which participants would prefer to receive teleconsultation advice. About 77% said they would use it for new complaint counseling, 75.1% for modifying prescription dosage, and 55.6% for infection-related advice.

**Figure 3:**
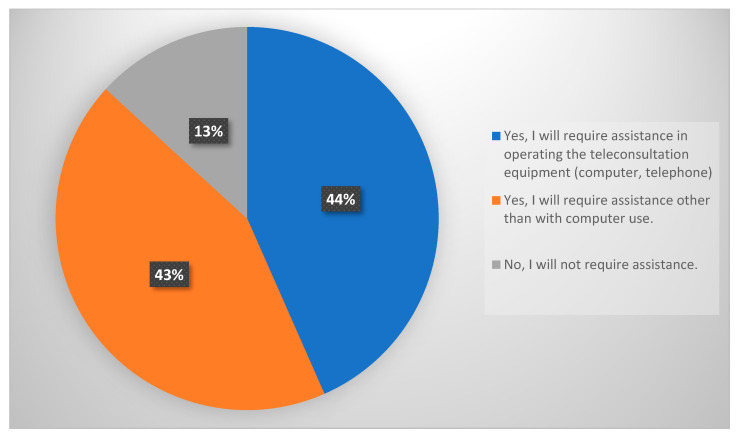
Participants’ need for assistance to participate in a teleconsultation (n = 189).

When asked if they would require assistance to engage in a teleconsultation, 44% of the respondents indicated that they would need assistance operating the teleconsultation equipment (computer, telephone), 43% would require support with teleconsultation beyond computer use, while 13% would not require assistance, as shown in Figure 3.

**Figure 4:**
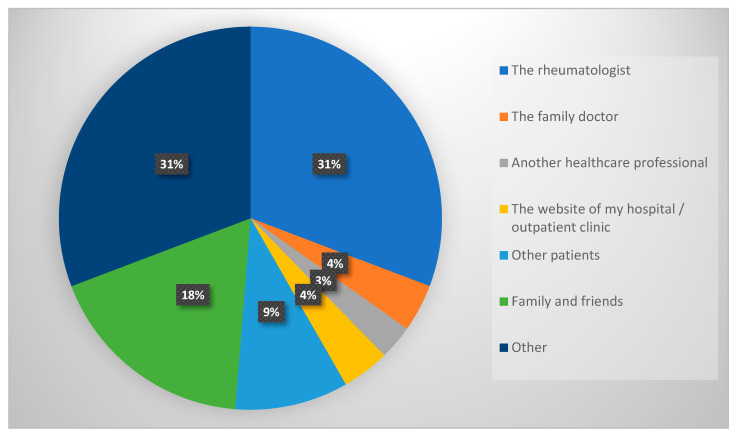
The source of information about the rheumatology teleconsultations among participants (n = 189).

Participants’ sources of knowledge on rheumatology teleconsultations are depicted in Figure 4. Among many sources, the rheumatologist was the primary source of information for our study participants.

**Figure 5:**
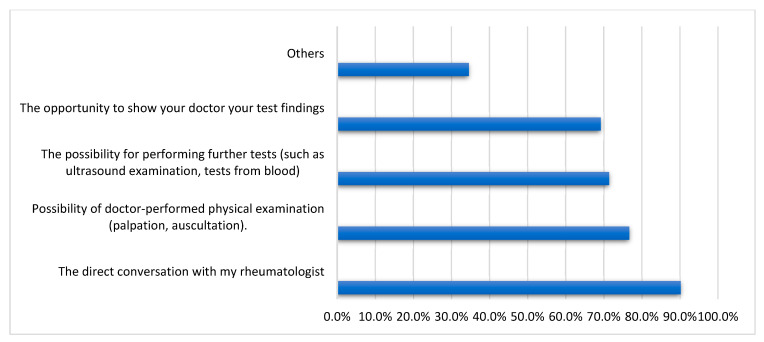
What participants appreciate most about direct contact with the rheumatologist during traditional outpatient clinic consultations in telemedicine non-acceptant group (n = 133).

As shown in Figure 5, when we asked the non-acceptant group what they liked most about direct contact during traditional OPC visits, 90.2% said they prefer direct conversation with their rheumatologist, 76.7% prefer a physical examination, and 69.2% said direct contact with the doctor is an opportunity to show him/her test results.

**Figure 6:**
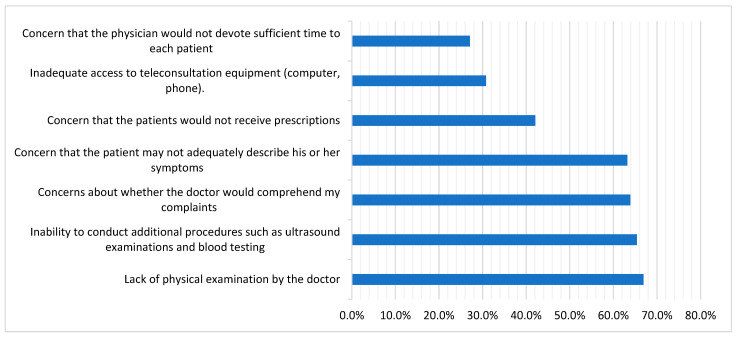
Concerns associated with teleconsultations with rheumatologist, stated by the teleconsultation non-acceptant group (n = 133).

Figure 6 highlights the concerns related to rheumatology teleconsultations that were expressed by the teleconsultation non-acceptant group.

Walking difficulties (76.8%), limited transport options to the rheumatology OPC (73.2%), and fear of becoming infected (73.2%) were the main responses we received from the acceptant group when we asked them why they planned to continue receiving teleconsultations even after the COVID-19 pandemic, as illustrated in Table 4.

## 4. Discussion

We conducted this study to evaluate the attitudes and perspectives of Egyptian patients with ARDs regarding the use of TM for disease follow-up because this is a collaborative decision-making process between patients and rheumatic healthcare providers.

The 189 ARD patients who participated in the study were divided into two groups based on their acceptance of teleconsultation: the non-acceptant group (70.4%) and the acceptant group (29.6%). There was a significant difference between the two groups in terms of educational level, chronic kidney and heart disease, and administration of hydroxychloroquine. However, there was no significant difference regarding distance from OPC, frequency of rheumatologist visits, exemption from insurance copayments, or prior COVID-19 infection. Approximately half of the individuals utilized virtual rheumatology consultations during the COVID-19 pandemic, predominantly by WhatsApp and phone. Approximately 87% would require assistance in operating the teleconsultation, either in terms of technology (computer, telephone) or beyond. Among various sources, the rheumatologist was the key source of knowledge on teleconsultation. The preference for direct conversation with the rheumatologist and the need for physical examination were the main barriers to teleconsultation.

In fact, there are undeniable advantages to the spread of TM, which can serve as an extra and possibly useful tool for patient diagnosis, treatment, rehabilitation, and follow-up monitoring [21]. Furthermore, decreasing the number of hospital visits not only decreases travel time and associated stress, but also promises financial benefits by lowering costs for both patients and healthcare payers [22]. So and colleagues [13] carried out an online survey on 103 patients diagnosed with lupus nephritis and found that 57.4% of respondents were willing to undergo TM follow-up. Cavagna et al. studied 137 patients with rheumatic disorders early in the COVID-19 pandemic and found that 137 (78%) preferred a TM visit to an in-person visit [12]. However, we found a low rate of TM acceptance (29.6%) in our Egyptian cohort. In Australia, a telerheumatology clinic was established, and questionnaires were conducted to evaluate patients’ points of view regarding the telerheumatology encounter. Additionally, a subsample of patients took part in focus groups to investigate the acceptance of the service. Patients are comfortable using telerheumatology, which involves the use of videoconferencing, for follow-up care of patients who already have an established disease [23]. However, in another study conducted in the United Kingdom, it was shown that 86% of patients with ARDs expressed a negative perception of TM in comparison to face-to-face consultations, specifically in terms of assessment accuracy [24].

There was a significant difference between the TM acceptant and non-acceptant participants in terms of educational level. Cavagna and colleagues have found that persons who lived further away and had a higher level of education were more willing to accept TM in lieu of an in-person visit [12]. Additionally, another study on 469 patients from Spain found that patient satisfaction with telerheumatology was connected to their level of education [25]. It was reported that acceptance of TM is inversely associated with a diagnosis of undifferentiated connective tissue disease [12].

During the COVID-19 pandemic, about half of our participants used virtual rheumatology consultations, mainly WhatsApp and phone consultations. WhatsApp is a popular messaging tool for communicating with people and groups. WhatsApp makes up a significant proportion of daily smartphone usage [26]. It is a helpful tool for TM since it has a large subscriber base, offers free services, and encrypts conversations and chats end-to-end [27]. In addition, WhatsApp enables the evaluation of radiological images sent by patients. However, there are drawbacks to using the WhatsApp platform in comparison to the standard teleconsultation platforms in terms of patient data protection, security, and legislation [27].

In fact, teleconsultation has limited use in Egypt. This could be owing to the lack of guidelines. However, during the COVID-19 pandemic, TM was used to continue care [28]. Regarding advice through teleconsultation, about 77% of our responders said they would use it for new complaint counseling. According to European Alliance of Associations for Rheumatology (EULAR) data on remote care, TM has been found to be feasible for the online management of patients with rheumatoid arthritis and assessment of COVID-19 infection in a cohort of patients with SLE [29,30]. The vast majority of studies have been conducted on noninflammatory rheumatic disorders, with OA being the disease for which the majority of telehealth interventions have been designed. Most interventions for inflammatory rheumatic disorders have been designed for RA patients.

TM is a vast field that involves a wide range of technology and strategies for providing care at a distance [31]. Approximately 87% of our respondents reported that they would require assistance using the teleconsultation equipment (computer, telephone) or beyond. This is a considerable hurdle for TM generalization amongst Egyptian individuals with ARDs.

Among many sources, the rheumatologist was the primary source of TM information for the study participants. Several studies have demonstrated increased quality of care when a rheumatologist is included in the care and monitoring of patients with rheumatic diseases, emphasizing the importance of rheumatologists in the health education, treatment, and follow-up of patients with rheumatic diseases [32,33]. Rheumatologists have been compelled to quickly adopt and adapt to TM. Limited clinic capacity, high-risk patient populations, and rheumatologists being redeployed to other tasks have significantly restricted access to care, especially in urban regions [34].

Despite its many advantages, TM is still not commonly used in the treatment of rheumatic and musculoskeletal diseases [16]. Persistent hurdles appear to be impeding the long-term implementation of TM [16]. In our cohort, the TM non-acceptant participants have many concerns regarding TM, such as the inability to conduct additional procedures such as ultrasound, lack of physical examination, and concern that the patient may not adequately describe his/her symptoms. In a Polish study, 244 rheumatology patients were questioned about their attitudes toward TM; the most often expressed concerns about virtual consultation were the inability to undergo extra tests and the inability to be physically evaluated by rheumatologists [35]. According to a survey of rheumatologists, 19% of patients are unsuitable for TM due to diagnostic ambiguity or disease complexity [36].

The inability to undergo physical examinations was the most commonly stated impediment to TM [35]. Several major impediments to widespread adoption have been identified, including patients’ and providers’ lack of knowledge of the possibilities, lack of comfort with TM technologies, patient identification and privacy concerns, and reimbursement limitations [37,38]. In our cohort, direct conversation with the rheumatologist and physical examination were preferred among the TM non-acceptant group. So, TM visits can be used to conduct an in-depth triage to evaluate whether an urgent in-person evaluation is required [34].

Walking difficulties, limited transport options to the rheumatology OPC, and fear of becoming infected were the main responses we received from the TM acceptant group when we asked them why they planned to continue receiving teleconsultations even after the COVID-19 pandemic. TM can improve healthcare efficiency, reduce travel distances, and improve access to healthcare services [39]. Significant uncertainty exists over TM payment rates following the pandemic, which will undoubtedly have an impact on the possibilities for further deployment and expansion of virtual care [34].

In fact, during the COVID-19 lockdown, TM increased from 10% to 90% of patient contacts in some settings in just one week [40,41]. The respondents’ experience with TM has altered their perspectives, and many of them would urge continuing its usage in the future, particularly with patients who have stable chronic inflammatory disease [42].

This study has many strengths. First, this study sheds light on the potential barriers to and drivers of teleconsultation deployment among Egyptian patients with ARDs. Second, this study provides critical information on the actual usage of telerheumatology during the COVID-19 pandemic.

Our study has a number of limitations. First, this study was conducted on Egyptian patients and hence may not be generalizable to other regions of the world. The second limitation is the absence of follow-up. Nonetheless, the aim of the present study was to assess the immediate feasibility and patient responses. Planned follow-up can compare the outcomes of patients who chose teleconsultation with those of patients who did not. Third, because a convenience sample approach was adopted, selection bias may have occurred.

## 5. Conclusions

In conclusion, our survey results suggest that the majority of Egyptian patients with rheumatic diseases are opposed to receiving rheumatology assistance via information and communication technology. They are concerned about teleconsultation because it does not include a physical examination by their doctors, and they will be unable to undergo further procedures such as US and blood testing. The majority of Egyptian patients with ARDs require assistance in using teleconsultation technology, which is the most significant barrier to the spread of TM. Recognizing and dealing with concerns at all levels is critical for raising teleconsultation awareness. To promote teleconsultation practice, we recommend that this study be replicated utilizing a qualitative research approach.

## Figures and Tables

**Table 1:** Sociodemographic data of the study patients with autoimmune and rheumatic diseases (n = 189).

Variable Mean ± SD, n (%)	Total (n = 189)	Non-Acceptant Group (n = 133)	Acceptant Group (n = 56)	*p*
Gender				0.320
Female	167 (88.4)	115 (86.5)	52 (92.9)	
Male	22 (11.6)	18 (13.5)	4 (7.1)	
Age (years)	37.41 ± 11.71	36.77 ± 10.82	38.95 ± 13.75	0.289
Marital status				0.755
Single	41 (21.7)	31 (23.3)	10 (17.9)	
Widow	7 (3.7)	5 (3.8)	2 (3.6)	
Divorced	7 (3.7)	4 (3.0)	3 (5.4)	
Married	134 (70.9)	93 (69.9)	41 (73.2)	
Residence				0.910
Rural	63 (33.3)	44 (33.1)	19 (33.9)	
Urban	126 (66.7)	89 (66.9)	37 (66.1)	
Socioeconomic status				0.316
Low	30 (15.9)	20 (15.0)	10 (17.9)	
Intermediate	154 (81.5)	108 (81.2)	46 (82.1)	
High	5 (2.6)	5 (3.8)	0	
Education level				0.001 *
No formal education	13 (6.9)	4 (3.0)	9 (16.1)	
Primary/secondary school	7 (3.7)	2 (1.5)	5 (8.9)	
High School	48 (25.4)	38 (28.6)	10 (17.9)	
Graduate	96 (50.8)	70 (52.6)	26 (46.4)	
Postgraduate	25 (13.2)	19 (14.3)	6 (10.7)	
Employment				0.501
Employed	66 (34.9)	50 (37.6)	16 (28.6)	
Retired	5 (2.6)	4 (3.0)	1 (1.8)	
Out of work	98 (51.9)	67 (50.4)	31 (55.4)	
Still a student	20 (10.6)	12 (9.0)	8 (14.3)	
Smoking habit				0.502
Smoker	7 (3.7)	6 (4.5)	1 (1.8)	
Former smoker	7 (3.7)	4 (3.0)	3 (5.4)	
Never	175 (92.6)	123 (92.5)	52 (92.9)	

* *p* < 0.05.

**Table 2:** Clinical and therapeutic data of the study patients with autoimmune and rheumatic diseases (n = 189).

Variable Median (Min–Max) n (%)	Total (n = 189)	Non-Acceptant Group (n = 133)	Acceptant Group (n = 56)	*p*
Diagnosis				0.304
Osteoarthritis	30 (15.9)	17 (12.8)	13 (23.2)	
Psoriatic arthritis	1 (0.5)	1 (0.8)	0	
Systemic lupus erythematosus	39 (20.6)	33 (24.8)	6 (10.7)	
Rheumatoid arthritis	45 (23.8)	29 (21.8)	16 (28.6)	
Sjogren syndrome	2 (1.1)	2 (1.5)	0	
Ankylosing spondylitis	8 (4.2)	7 (5.3)	1 (1.8)	
Idiopathic inflammatory myositis	13 (6.9)	9 (6.8)	4 (7.1)	
Gout	5 (2.6)	3 (2.3)	2 (3.6)	
Systemic sclerosis	6 (3.2)	4 (3.0)	2 (3.6)	
Juvenile idiopathic arthritis	1 (0.5)	0	1 (1.8)	
Vasculitis	2 (1.1)	1 (0.8)	1 (1.8)	
Others	37 (19.6)	27 (20.3)	10 (17.9)	
Disease duration (months)	48 (1–360)	48 (1–360)	38 (2–324)	0.651
Self-reported disease severity (past week)				0.306
Under control	12 (6.3)	6 (4.5)	6 (10.7)	
Mild	44 (23.3)	31 (23.3)	13 (23.2)	
Moderate	88 (46.6)	61 (45.9)	27 (48.2)	
Severe	45 (23.8)	35 (26.3)	10 (17.9)	
Comorbidities				
Hypertension	60 (31.7)	44 (33.1)	16 (28.6)	0.543
Lung disease	30 (15.9)	22 (16.5)	8 (14.3)	0.698
Diabetes mellitus	18 (9.5)	14 (10.5)	4 (7.1)	0.469
Kidney disease	21 (11.1)	20 (15.0)	1 (1.8)	0.008 *
Heart disease	19 (10.1)	18 (13.5)	1 (1.8)	0.014 *
Malignancy	3 (1.6)	3 (2.3)	0	-
Medications				
Corticosteroids	98 (51.9)	72 (54.1)	26 (46.4)	0.333
csDMARDs				
Methotrexate	45 (23.8)	30 (22.6)	15 (26.8)	0.533
Hydroxychloroquine	93 (49.2)	72 (54.1)	21 (37.5)	0.037 *
Mycophenolate mofetil	30 (15.9)	22 (16.5)	8 (14.3)	0.698
Leflunomide	37 (19.6)	26 (19.5)	11 (19.6)	0.988
Biologic or targeted DMARDs				
TNF-alpha inhibitors	17 (9.0)	14 (10.5)	3 (5.4)	0.404
Interleukin-6 inhibitors	5 (2.6)	3 (2.3)	2 (3.6)	0.634
Rituximab	9 (4.8)	7 (5.3)	2 (3.6)	1
Interleukin-17 inhibitors	8 (4.2)	6 (4.5)	2 (3.6)	1
Interleukin-1 inhibitors	2 (1.1)	1 (0.8)	1 (1.8)	0.506
JAK inhibitors	3 (1.6)	2 (1.5)	1 (1.8)	1
Others	122 (64.6)	87 (65.4)	35 (62.5)	0.702

* *p* < 0.05; csDMARDs: conventional synthetic disease-modifying antirheumatic drugs.

**Table 3:** Healthcare services for study patients with autoimmune and rheumatic diseases (n = 189).

Variable Mean ± SD, Median (Min–Max) n (%)	Total (n = 189)	Non-Acceptant Group (n = 133)	Acceptant Group (n = 56)	*p*
Distance from the outpatient clinic				0.461
Living in the same province	16 (8.5)	13 (9.8)	3 (5.4)	
In another province within the same region	20 (10.6)	16 (12.0)	4 (7.1)	
Living in another region	148 (78.3)	100 (75.2)	48 (85.7)	
Being accompanied to the hospital	5 (2.6)	4 (3.0)	1 (1.8)	
Frequency of visits to rheumatologist in the last year				0.227
Every < 4 months	105 (55.6)	72 (54.1)	33 (58.9)	
Every 4–6 months	34 (18.0)	28 (21.1)	6 (10.7)	
Every > 6 months	50 (26.5)	33 (24.8)	17 (30.4)	
Exemption from insurance copayments				0.940
Complete	38 (20.1)	27 (20.3)	11 (19.6)	
Partial	31 (16.4)	21 (15.8)	10 (17.9)	
No exemption	120 (63.5)	85 (63.9)	35 (62.5)	
Prior infection with COVID-19	96 (50.8)	72 (54.1)	24 (42.9)	0.157
Virtual rheumatology appointment during the COVID-19 pandemic	96 (50.8)	71 (53.4)	25 (44.6)	0.272

**Table 4:** Reasons to continue teleconsultations at the rheumatology outpatient clinic following the SARS-CoV-2 pandemic among acceptant group (n = 56).

Variable	Acceptant Group n (%)
Walking difficulties	43 (76.8)
Transport options to the rheumatology outpatient clinic are limited	41 (73.2)
Fear of becoming infected	41 (73.2)
Too long of a wait for an appointment	43 (76.8)
The potential for reducing wait times for outpatient clinic appointments	47 (83.9)

## Data Availability

The datasets used and/or analyzed during the current study are available from the corresponding author upon reasonable request.

## Abbreviations

ARDs	autoimmune and rheumatic diseases
COVID-19	coronavirus disease 2019
EULAR	European Alliance of Associations for Rheumatology
OA	osteoarthritis
OPC	outpatient clinic
RA	rheumatoid arthritis
SLE	systemic lupus erythematosus
TM	telemedicine
WHO	World Health Organization
